# Coffee Intake Is Associated with a Lower Liver Stiffness in Patients with Non-Alcoholic Fatty Liver Disease, Hepatitis C, and Hepatitis B

**DOI:** 10.3390/nu9010056

**Published:** 2017-01-10

**Authors:** Alexander Hodge, Sarah Lim, Evan Goh, Ophelia Wong, Philip Marsh, Virginia Knight, William Sievert, Barbora de Courten

**Affiliations:** 1 Gastroenterology and Hepatology Unit, Monash Health, Melbourne 3168, Australia; virginia.knight@monash.edu (V.K.); william.sievert@monash.edu (W.S.); 2 Centre for Inflammatory Disease, School of Clinical Sciences, Monash University, Melbourne 3168, Australia; 3 School of Clinical Sciences, Monash University, Melbourne 3168, Australia; sarah146lim@gmail.com (S.L.); ektgoh@outlook.com (E.G.); Ophelia.Wong@monashhealth.org (O.W.); philmarsh11@gmail.com (P.M.); 4 Monash Centre for Health, Research and Implementation, School of Public Health and Preventive Medicine, Monash University, Melbourne 3168, Australia; barbora.decourten@monash.edu

**Keywords:** liver disease, coffee, functional foods, elastography

## Abstract

There is emerging evidence for the positive effects or benefits of coffee in patients with liver disease. We conducted a retrospective cross-sectional study on patients with non-alcoholic fatty liver disease (NAFLD), hepatitis C virus (HCV), and hepatitis B virus (HBV) infection to determine the effects of coffee intake on a non-invasive marker of liver fibrosis: liver stiffness assessed by transient elastography (TE). We assessed coffee and tea intake and measured TE in 1018 patients with NAFLD, HCV, and HBV (155 with NAFLD, 378 with HCV and 485 with HBV). Univariate and multivariate regression models were performed taking into account potential confounders. Liver stiffness was higher in males compared to females (*p* < 0.05). Patients with HBV had lower liver stiffness than those with HCV and NAFLD. After adjustment for age, gender, smoking, alcohol consumption, M or XL probe, and disease state (NAFLD, HCV, and HBV status), those who drank 2 or more cups of coffee per day had a lower liver stiffness (*p* = 0.044). Tea consumption had no effect (*p* = 0.9). Coffee consumption decreases liver stiffness, which may indicate less fibrosis and inflammation, independent of disease state. This study adds further evidence to the notion of coffee maybe beneficial in patients with liver disease.

## 1. Introduction

Coffee is second only to water as the world’s most popular beverage and has been consumed for many centuries. It is thought that 2.25 billion cups of coffee are consumed every day worldwide, and this number continues to grow [1]. Coffee is a complex mixture of proteins, carbohydrates, lipids, proteins, potassium, magnesium, antioxidants, and caffeine [2]. Coffee has been suggested to have health benefits in a variety of diseases [3]. There is limited but emerging evidence that coffee lowers risk of several cancers including colorectal, liver, renal, ovarian, pancreatic, oesophageal, endometrial, and pharyngeal cancer [3]. Once thought to be related to an increased risk of cardiovascular disease and hypertension, recent prospective studies and meta-analyses demonstrate a beneficial effect [4]. Coffee consumption also results in a lower risk of developing type 2 diabetes [5] and metabolic syndrome [6], and prevents gestational diabetes [7]. Moderate coffee intake may also reduce the risk of neurodegenerative conditions such as Parkinson’s Disease and Alzheimer’s disease [8,9]. Importantly, a recent meta-analysis of 17 prospective studies showed that light to moderate coffee intake was associated with a reduction of all-cause mortality [10].

There is emerging evidence for a hepatoprotective effect of coffee in a wide range of liver conditions of different severity ranging from elevation of liver enzymes to fibrosis and hepatocellular carcinoma [11]. In addition, in both healthy populations and those at risk of liver disease, coffee drinkers have lower aminotransferases and gamma-glutamyl transferase levels compared to their non-coffee drinking counterparts [12,13].

Although liver biopsy is still considered the gold standard for the diagnosis and staging of both fibrosis and steatosis, it is expensive, invasive, and associated with a variety of risks and morbidity [14]. Additionally, with the increasing prevalence of non-alcoholic fatty liver disease (NAFLD), it is not practical or cost-effective to perform liver biopsies in those suspected of having fibrosis. Therefore, non-invasive methods to assess liver fibrosis and steatosis are gaining in popularity. Transient elastography (TE) using FibroScan^®^ (EchoSens, Paris, France) is the most widely used and studied method, having considerable accuracy and reproducibility. It is regarded as a surrogate for liver biopsy for grading liver fibrosis in patients with chronic liver disease [15] and more recently has been used to assess the presence and stage of steatosis in the liver with good accuracy by measuring the degree of ultrasound attenuation, a reading called the controlled attenuation parameter (CAP) [16].

In the present study, we sought to determine if there is an association between coffee intake and liver stiffness as assessed by transient elastography in patients with NAFLD, chronic hepatitis C (HCV), and chronic hepatitis B (HBV) infections.

## 2. Methods

### 2.1. Study Population and Design

We analysed retrospectively cross-sectional data from patients with HCV, HBV, or NAFLD attending the TE liver clinic at Monash Medical Centre (Melbourne, Australia) between May 2012 and November 2013. HCV and HBV infection were confirmed by detectable HCV viral load or hepatitis B surface antigen, respectively. No HCV patients were receiving anti-viral therapy during the study period. NAFLD patients were referred from the community or from our outpatient gastroenterology clinic. Diagnosis of NAFLD was made (by a radiologist) on the basis of ultrasound [17] and after the exclusion of other liver diseases.

### 2.2. Data Collection

Self-reported coffee, tea, and alcohol drinking habits were collected as part of a standard history taking during a consultation at the TE liver clinic by one of two gastroenterologists who also performed TE. A single cup of coffee was defined as a single heaping teaspoon of instant coffee, a single Nespresso^®^ (Nestlé, Lausanne, Switzerland) pod or 30 mL of espresso coffee. A cup of boiled coffee was considered equivalent to one heaping teaspoon of very finely ground coffee beans. A cup of filtered coffee (pour over) was approximately 30 g or 2–4 Tbsp of ground coffee. The number of cups of tea corresponded to the number of tea bags (or equivalent leaf tea). Daily alcohol intake in grams was also determined. We also collected information on coffee and tea type, smoking habits (yes or no), body weight, body mass index, gender, and age. TE was assessed using FibroScan^®^ (Echosens, Paris, France) M or XL probe with the latter available in early 2013. Liver stiffness measurement (LSM) was assessed for all patients, and CAP was reported when available for the XL probe in early 2013. All Fibroscan^®^ studies were performed with participants having fasted for at least 2 h. Only LSM and CAP studies with ≥10 valid readings and median measurement with interquartile range/median value ≤30% were analysed.

### 2.3. Statistical Analyses

Statistical analyses were performed using SAS JMP 12.1 Statistics Software (SAS Institute Inc., Cary, NC, USA). All statistical tests were two-sided with α = 0.05 and unpaired *t*-tests were performed to assess a difference between the genders. For categorical variables, percentages were displayed and continuous variables were displayed as mean and standard deviation. A chi-square test was used to assess differences in categorical variables. Differences between disease states were assessed by ANOVA with post-hoc Tukey analyses. Multivariate regression models were fitted to the dependent variables: liver stiffness and CAP. Daily coffee consumption was investigated for ≥2 cups per day. Multivariate regression models were first fitted to the following covariates of interest: age, gender, BMI, smoking status (yes, no), and alcohol consumption (categorised) and exam type.

## 3. Results

### 3.1. Patient Characteristics

A total of 1018 patients (155 patients with NAFLD, 378 with HCV and 485 with HBV) with available data on age, gender, liver stiffness, BMI, smoking status, and alcohol use was assessed between May 2012 and November 2013.

The characteristics the whole group and divided by gender are presented in Table 1. Overall patients were overweight (BMI > 25 kg/m^2^), with males having higher BMI. Median liver stiffness measurements were above the accepted normal cut-off of 5.5 kPa [18,19] with a higher stiffness in males. The overall CAP results were below the cut-off value suggested for the detection of significant steatosis [20]. One quarter of the sample smoked with higher smoking rates in males. Males drank more alcohol though daily consumption was low. One third of the cohort drank 2 or more cups of coffee per day.

Characteristics divided by disease state (NAFLD, HCV, and HBV) are presented in Table 2. Patients with HBV were younger and had lower weight, BMI, liver stiffness, and CAP values compared to the patients with HCV and NAFLD. They also drank less alcohol and coffee, but they had higher smoking rates compared to the patients with HCV and NAFLD. HBV patients had either inactive infection not requiring antiviral treatment or were taking anti-virals (*n* = 58) and had low stable or undetectable viral loads. Those with active HBV infection and elevated liver enzymes are not routinely referred for FibroScan due to the confounding effects active hepatitis has on liver stiffness. Patients with NAFLD were older, had the highest weight and BMI, had liver stiffness and, as expected, the highest CAP values. The mean CAP value was above the optimal cut-off of 255 dB/m for detection of Grade 2 steatosis [20]. Patients with NAFLD also drank more alcohol compared to patients with HBV and drank more coffee than patients with HBV but less than patients with HCV. Forty-six percent of the patients with HCV, 38% of patients with NAFLD and only 21% of HBV patients drank ≥2 cups of coffee daily. Only 50% of patients had coffee types recorded with 72% drinking instant, 24% espresso-based, 2% filtered, 1% boiled, and 1% decaffeinated instant.

### 3.2. Relationship between Coffee and Tea Consumption and Liver Stiffness

After adjustment for age, gender, BMI, smoking, alcohol consumption (categorised ≥ 30 g day), examination type (M or XL probe), and disease state, those individuals who drank ≥2 cups of coffee per day had lower liver stiffness compared to those individuals who drank <2 cups of coffee (Table 3). The interaction between coffee consumption and disease state in this model was not significant (*p* = 0.7), suggesting that coffee consumption is associated with lower liver stiffness in all patients including those with NAFLD, HCV, and HBV. In addition, the interaction between exam type (M or XL) and coffee consumption was also not significant (*p* = 0.2).

After adjustment for age, gender, BMI, smoking, alcohol consumption (categorised ≥ 30 g·day), examination type, and disease state, drinking 2 or more cups of tea per day (*p* = 0.9) or total tea cups per day (*p* = 0.8) was not associated with liver stiffness.

### 3.3. Relationship between Coffee Consumption and CAP

After adjustment for age, gender, BMI, and alcohol consumption, CAP was not associated with coffee consumption (*p* = 0.7) or tea consumption (*p* = 0.8). There was no significant interaction between cups of coffee and groups (HBV, HCV, or NAFLD); therefore, no sub-group analysis was performed.

## 4. Discussion

Here we show for the first time an association between coffee intake and liver stiffness assessed by transient elastography in three common liver diseases. Our study found that coffee intake at 2 or more cups per day was associated with lower liver stiffness, a non-invasive marker of liver fibrosis in patients with NAFLD, HBV, and HCV. While this was a retrospective cross-sectional study, it adds to the growing body of literature demonstrating the hepatoprotective benefits of coffee. We found no association between tea consumption and liver stiffness.

Previous studies have demonstrated benefits associated with coffee in patients with HCV and NAFLD [21,22], with the strongest evidence of benefit in those with hepatitis C-related liver disease. Khalaf et al. found that caffeine, mostly from coffee, was protective against advanced fibrosis using the FibroSURE test, a serum panel of non-invasive markers of fibrosis [21]. Coffee intake of 3 or more cups in HCV-infected individuals has been associated with lower rates of disease progression [23] and with an improved response to peginterferon/ribavirin therapy [24]. Mechanisms suggested include inhibition of the propagation of HCV virus [25], a reduction in oxidative damage, serum markers of collagen synthesis, and increased telomere length, which may account for the reduction in disease progression and HCC development [26].

A protective role for coffee consumption in NAFLD is controversial. Some studies have shown that, in those with NAFLD, coffee consumption may be protective against liver steatosis and fibrosis [27,28]. Ultrasound-based studies have shown coffee intake to be inversely associated with the degree of liver steatosis [27]. Similarly, studies using serum markers of fibrosis and histology have shown coffee intake inversely associated with fibrosis in NAFLD [22,29,30]. Molloy et al. was one of the first to show a correlation with coffee consumption and liver histopathology [29]. They showed higher coffee consumption in individuals with steatosis compared to those with non-alcoholic steatohepatitis (NASH), suggesting a protective effect against inflammation. Furthermore, in those with NASH, coffee intake was associated with a significant reduction in risk of advanced fibrosis [29]. However, a recent prospective study found no protective effect of coffee on the development of liver steatosis [22]. Moreover, a community-based study of 1223 people found no association between coffee intake and the prevalence of fatty liver [31]. We also looked at the association between liver steatosis and coffee intake using a non-invasive marker, CAP, which has been reported on our FibroScan^®^ assessments since early 2013. Consistent with other studies using ultrasonographic methods [22,31] we did not show a relationship between coffee consumption and CAP. These results may be due to the patient numbers required to provide sufficient power to determine a significant difference. In addition, many patients with HBV or HCV do not have liver steatosis. HBV and HCV patients in our study had a mean BMI 24 kg/m^2^ and 26 kg/m^2^, respectively, and therefore were less likely to have steatosis compared to people with NAFLD who had a BMI of 29 kg/m^2^. Lending credence to this was the lower CAP readings in HBV and HCV patients. HBV and HCV patients had CAP readings of 210 dB/m and 202 dB/m, respectively, below the proposed cut-off of 232 dB/m for detection of significant steatosis (Grade 1) [20]. Only the CAP values in NAFLD (261 dB/m) were suggestive of liver steatosis. By measuring the degree of ultrasound attenuation through the liver, the CAP reading provides good sensitivity and specificity for detection of steatosis [32,33]. The results are not affected by disease aetiology (HCV, HBV, or NAFLD) [34]. CAP is non-invasive, reproducible, inexpensive, and can be determined at the same time liver stiffness measurements are obtained.

Our findings demonstrate an association between coffee and liver stiffness in patients with HBV infection. To date, evidence demonstrating the benefits of coffee in HBV-induced liver disease has been limited. Studies have shown mixed results regarding whether coffee reduces the risk of HBV-associated hepatocellular carcinoma [35,36]. There has only been one study of hepatitis B patients investigating the association of caffeine consumption with liver fibrosis. In that study, all caffeinated beverages, rather than coffee specifically, were examined and caffeine had no effect on liver stiffness using FibroScan^®^ [37].

A strength of our findings is in the diversity of the participant population. Our clinic is representative of most tertiary referral centres across Australia with a wide diversity in ethnicity and socio-economic standing. The use of transient elastography allows us to study all patients referred to our clinic who would have a broad range of disease severities, whereas studies using histology for fibrosis and steatosis may have a selection bias towards those with advanced or more active disease. We did not have access to liver histology to correlate with FibroScan^®^ findings. Data on liver enzymes such as serum alanine transaminase (ALT) at the time of FibroScan^®^ would have also been useful. In particular, in HBV infection, a flare with ALT elevation can impact the transient elastography results by artificially raising the LSM [38,39]. In HCV, an ALT greater than twice the upper limit of normal can also influence transient elastography results [40]. It is possible that the lower liver stiffness results we observed in coffee drinkers reflects a combination of less liver inflammation and fibrosis. A systematic review of coffee’s impact on patients with liver disease has shown that coffee improves liver inflammation with a reduction in liver enzymes [41]. Findings in a rat model of NASH indicates that this may occur through reduced liver concentrations of the pro-inflammatory cytokines tumour necrosis factor alpha and transforming growth factor β (TGF-β), with an increase in anti-inflammatory cytokines interleukin-4 and interleukin-10 [42]. Studies involving rodent models of chronic liver disease have proposed mechanisms on how coffee improves liver fibrosis. Animals given coffee had reduced levels of fibrosis and TGF-β, a potent driver of liver fibrosis [43,44,45]. Other fibrogenic cytokines such as platelet-derived growth factor (PDGF)-β and connective tissue growth factor (CTGF) are also reduced in these fibrotic models when administered coffee [44,46]. TGF-β, PDGF-β, and CTGF activate hepatic stellate cells (HSC), which express collagen and are involved in liver fibrosis progression. In murine models of fibrosis, coffee reduces HSC activation as demonstrated by a reduction in alpha-smooth muscle actin. These studies suggest that coffee has an effect on profibrotic cytokines and stellate cells with a resultant reduction in fibrosis. In a rat model of NASH, coffee drinking improved both fibrosis and inflammation through the induction of both hepatic endoplasmic reticulum and mitochondrial chaperones, thereby increasing expression of antioxidant proteins [47]. Ideally, future studies should include liver biopsy in those with advanced liver disease to correlate histological changes in fibrosis and inflammation with FibroScan results.

The degree to which the beneficial effects of coffee are due to its metabolites or to caffeine is not clear. Caffeine can reduce liver inflammation and fibrosis in animal models through its effect on hepatic stellate cells [48] and hepatocytes [49]. Other studies of de-caffeinated coffee suggest a role for factors other than caffeine in hepatoprotection, with animals fed a high fat diet and de-caffeinated coffee having less liver steatosis, fibrosis, and inflammation than the controls [42]. In addition to caffeine, there are over 1000 different constituents in coffee and the extraction method can alter their concentration. One such potential candidate for beneficial liver-related effects is chlorogenic acid. In animal studies, chlorogenic acid attenuates liver steatosis [50] and reduces inflammation in NASH [42]. The concentration of chlorogenic acid varies with different coffee preparation methods and is higher in boiled versus filtered coffee [51]. Future studies should focus on determining which coffee extraction or preparation methods enhance constituents with the greatest liver-related benefits.

## 5. Conclusions

In NAFLD, HCV and HBV associated liver diseases coffee consumption, not tea, was associated with lower liver stiffness, a non-invasive marker of liver fibrosis. Our study adds to the growing evidence that coffee consumption has a beneficial effect in a variety of liver diseases. However, we need prospective randomised controlled trials to determine whether coffee consumption will actively reduce liver fibrosis and inflammation. As our population ages, the prevalence of chronic liver disease will increase, very likely led by NAFLD. We may look to coffee as one arrow in the quiver of therapies and lifestyle approaches to tackle this problem.

## Figures and Tables

**Table 1:** Patient characteristics overall and by gender.

Variable	All (*n* = 1018)	Females (*n* = 441)	Males (*n* = 577)
Age (years)	48 ± 13	48 ± 13	47 ± 12
Weight (kg)	72 ± 16	64 ± 14	78 ± 14 *
BMI (kg/m^2^)	26 ± 4	25 ± 5	26 ± 4 *
Liver stiffness (kPa)	8.4 ± 7.7	7.6 ± 6.2	8.9 ± 8.6 *
CAP (dB/m) (*n* = 373 males, 264 females)	214 ± 54	202 ± 58	221 ± 50 *
Alcohol use (g)	4.9 ± 14.6	2.4 ± 9.6	6.7 ± 17.3 *
Coffee cups	1.3 ± 1.7	1.1 ± 1.5	1.5 ± 1.9 *
Coffee (≥2 cups·day, %)	33	27	37 *
Smoking (yes, %)	26	19	32 *

Variables are presented as mean ± SD for continuous variables. * *p* < 0.05 for *t*-test between the groups for continuous variables or chi-square for ordinal variables comparing males with females. Abbreviations: BMI: body mass index; CAP: controlled attenuation parameter (marker of steatosis).

**Table 2:** Patient characteristics divided by disease state (HBV, HCV, and NAFLD).

Variable	NAFLD (*n* = 155)	HCV (*n* = 378)	HBV (*n* = 577)
Age (years)	56 ± 12	48 ± 11 ^a^	46 ± 13 ^b,c^
Weight (kg)	82 ± 16	74 ± 15 ^a^	66 ± 13 ^b,c^
BMI (kg/m^2^)	29 ± 4	26 ± 4 ^a^	24 ± 4 ^b,c^
Liver stiffness (kPa)	10.7 ± 11.0	10.0 ± 8.6 ^a^	6.3 ± 4.5 ^b,c^
CAP (dB/m) (*n* = 104 NAFLD, 217 HCV, 316 HBV)	261 ± 57	210 ± 44 ^a^	201 ± 52 ^b^
Alcohol use (g)	5.5 ± 12.0	8.2 ± 20.5	2.0 ± 7.5 ^b,c^
Tea cups	1.3 ± 1.8	1.1 ± 1.7 ^a^	0.9 ± 1.5 ^b,^*
Coffee cups	1.4 ± 1.4	1.9 ± 2.2 ^a^	0.9 ± 1.2 ^b,c^
Coffee (≥2 cups·day, %)	38	46	21 *
Smoking (yes, %)	15	37	48 *

^a^ NAFLD vs. HCV, ^b^ NAFLD vs. HBV, ^c^ HCV vs. HBV using one-way ANOVA with post-hoc Tukey analyses. * Significance for chi-square analyses across the three groups. Abbreviations: BMI: body mass index; NAFLD: non-alcoholic fatty liver disease; HBV: hepatitis B virus infection; HCV: hepatitis C virus infection; CAP: controlled attenuation parameter (marker of steatosis).

**Table 3:** Multivariate analyses between liver stiffness and coffee consumption after adjustment for co-variates.

	Estimate	Std Error	T Ratio	Prob > (t)
Intercept	−0.49	0.36	−1.38	0.17
Age (years)	0.0062	0.0013	4.75	<0.0001
Gender (Males)	0.036	0.016	2.22	0.026
Examination type (Medium probe)	0.070	0.036	1.98	0.048
Log BMI (kg/m^2^)	1.47	0.24	6.1	<0.0001
Smoking (No/Yes)	0.1	0.041	2.42	0.0156
Alcohol (≥30 g·day)	0.023	0.068	0.35	0.7
Indication (NAFLD) vs HBV	0.58	0.032	1.8	0.072
Indication (HCV) vs. HBV	0.11	0.026	4.45	<0.0001
Coffee (≥2 cups·day)	−0.070	0.035	−2.02	0.0435

Abbreviations: BMI: body mass index, NAFLD: non-alcoholic fatty liver disease, HBV: hepatitis B virus infection, HCV: hepatitis C virus infection.
